# Editorial: Biomechanics, Sensing and bio-inspired control in rehabilitation and assistive robotics, volume II

**DOI:** 10.3389/fbioe.2026.1837753

**Published:** 2026-04-24

**Authors:** Jinghang Li, Keyi Wang, Changshen Li, Wujing Cao, Yi Liu, Liang Qiu

**Affiliations:** 1 College of Mechanical and Electrical Engineering, Harbin Engineering University, Harbin, China; 2 School of Mechatronical Engineering, Beijing Institute of Technology, Beijing, China; 3 Guangdong Provincial Key Lab of Robotics and Intelligent System, Shenzhen Institute of Advanced Technology, Chinese Academy of Sciences, Shenzhen, China; 4 Department of Radiation Oncology, Stanford University, Stanford, United States

**Keywords:** bio-inspired control, biomechanics, biosignal processing, human-robot interaction, rehabilitation robotics

## Introduction

The continuous evolution of modern rehabilitation and assistive robotics is driven by the seamless integration of biomechanics, multimodal sensing technologies, and bio-inspired control strategies. As the demand for personalized motor rehabilitation grows, researchers are moving beyond rigid, predefined robotic assistance toward paradigms that prioritize dynamic human-robot interaction and mechanical transparency. By leveraging advanced sensing modalities—such as high-density electromyography, computer vision, and dynamic force sensors—these systems can now decode complex human motor intentions. Coupled with bio-inspired control strategies that mimic natural neuromuscular coordination, these technologies optimize the mechanical compliance of assistive devices and significantly enhance clinical outcomes, fostering a more intuitive and patient-centric rehabilitation experience.

Building upon the successes of its predecessor, this Volume II of the Research Topic has collected 20 high-quality contributions that reflect the vibrant and multidisciplinary nature of this field. These 20 papers can be organized into four main focus areas: (1) Development and Evaluation of Rehabilitation and Assistive Robotics; (2) Human Motion Intention Recognition and Biosignal Processing; (3) Biomechanical Modeling and Neuromuscular Analysis; (4) Clinical Rehabilitation Strategies and Functional Training.

## Development and evaluation of rehabilitation and assistive rRobotics

This section highlights groundbreaking advancements in the design, control, and clinical evaluation of rehabilitation and assistive robotics. Focusing on wearable technologies, Huang et al. evaluated the efficacy and safety of a novel portable lumbar exoskeleton device featuring a coupled rigid-flexible parallel structure intended for patients with lumbar disc herniation. The device facilitated a combined conservative treatment approach by integrating multidirectional lumbar traction, six-degree-of-freedom range of motion training, and targeted resistance exercises. A multicenter randomized controlled trial demonstrated that the exoskeleton achieved comparable efficacy to traditional traction therapies, significantly improving pain relief and functional status while enhancing lumbar mobility. Ding et al. developed ChMER, a novel mobile exoskeleton robot integrated with an active body weight support walker designed specifically for early gait rehabilitation in young children with cerebral palsy. The system utilized an intrinsically compliant actuation paradigm employing quasi-direct drive-like joints and series elastic actuators, controlled via proprioceptive torque sensing and an assist-as-needed impedance control framework. Experimental validation confirmed that the robot provided high mechanical compliance and precise support force control, successfully enabling both stable passive walking and adaptive active rehabilitation training. Xu et al. evaluated a wearable hand orthosis for managing upper limb spasticity and promoting motor recovery in stroke survivors. Through a randomized controlled trial, participants used the orthosis to maintain an antispastic position for five hours daily alongside conventional therapy. Although wrist flexor spasticity was not significantly reduced, the intervention significantly improved upper extremity motor function, balance, and daily independence. These findings highlight a critical correlation between upper limb recovery and overall postural stability.

To optimize the dynamic interaction during such upper limb interventions, Li et al. mitigated patient muscle fatigue in active robotic rehabilitation via an adaptive port-Hamiltonian control strategy. This dual-layer architecture combined outer-loop admittance control with inner-loop energy shaping, utilizing real-time surface electromyography to map fatigue as a dissipative element. Experiments proved this fatigue-compensated approach preserved strict system passivity, successfully reducing muscle fatigue accumulation by 45% and extending training duration by 40% compared to fixed-parameter methods. In a similar pursuit of adaptive physiological support, Zhi et al. developed a wearable soft robotic system designed to provide safe and synchronized respiratory assistance using pneumatic origami actuators that apply compressive forces to the user’s abdomen. By establishing a thoracoabdominal biomechanical transmission model, the researchers implemented a dual-layer force-pressure coordinated control architecture guided by real-time respiratory flow and human-robot interaction force constraints. Clinical testing on healthy subjects confirmed that this interaction control strategy significantly enhanced key ventilation metrics, such as peak expiratory flow and minute ventilation, validating the system’s physiological adaptability and safety.

The scope of assistive robotics is further broadened by systems designed to augment activities of daily living and clinical caregiving. Li et al. engineered an intelligent bathing assistance system using a six-degree-of-freedom robotic arm equipped with an RGB-D camera and a rotating scrubber. The system utilizes YOLOv5s and a depth-correction algorithm for high-precision 3D localization, alongside cubic B-spline interpolation for adaptive scrubbing trajectories. Validation demonstrated robust skin detection, minimal localization errors, and smooth, surface-conforming movements achieved entirely without complex force-control algorithms. Focusing on nursing transfer tasks, Kurata et al. proposed a powered lifting device that assists patients from supine to sitting while intentionally preserving direct caregiver-patient physical touch. The dual-cable sling mechanism detects the caregiver’s implicit intentions (rotation, translation, or stay) through subtle cable tension variations to control its motors. Trials demonstrated an intention detection accuracy exceeding 70%, with users highly valuing the psychological comfort and intuitive postural adjustments afforded by maintained human-to-human contact. Finally, expanding robotic precision to traditional medicine, Li et al. developed a six-degree-of-freedom robotic acupuncture system to quantify and reproduce manual therapeutic techniques. The system records manual manipulations using computer vision and deep learning marker tracking, employing inverse kinematics for precise robotic trajectories and real-time force monitoring at the needle interface. Validation confirmed the robot replicated manual trajectories and force distributions exceptionally well. Furthermore, *in vivo* experiments demonstrated the system achieved significant analgesic effects comparable to master acupuncturists.

## Human motion intention recognition and biosignal processing

This section is dedicated to human motion intention recognition and the advanced processing of biosignals, highlighting how intelligent algorithms can decode complex human movements from various sensor modalities.

Focusing on non-invasive motion capture, He et al. developed a markerless 3D pose estimation method using monocular video for the early diagnosis of Parkinson’s disease. Integrating Transformer and Graph Convolutional Network architectures, their framework extracted spatiotemporal gait parameters, achieving a 93.3% prediction accuracy with a Random Forest classifier. Kamal et al. proposed a multi-modal framework using RGB and depth video to identify pain-indicative actions. Utilizing a deep neuro-fuzzy classifier on extracted spatial-temporal features, the system achieved classification accuracies up to 94.50% across benchmark datasets. Similarly targeting remote physical therapy, Ashraf et al. introduced a deep multimodal framework for the sensor-free biomechanical analysis of lower back pain exercises. The system fuses 3D body joint positions from depth data with 2D keypoints and semantic contours from synchronized RGB streams. By deploying a Transformer-based architecture to capture complex temporal motion patterns, the framework achieved state-of-the-art classification accuracies up to 94.73%. This approach offers a robust, scalable solution for automated patient monitoring in rehabilitation settings.

Transitioning to wearable sensors, Rafiq et al. developed a robust human locomotion and localization recognition system utilizing multimodal inertial, GPS, and audio signals from smartphones and wearables. The methodology employed Hamming windows and Butterworth filters for noise reduction, extracting diverse time-series features like Dynamic Time Warping. By applying a Fuzzy Entropy Classifier optimized via Yeo-Johnson transformation, the system achieved 90% and 91% accuracies on benchmark datasets, demonstrating strong generalization in uncontrolled, noisy environments.

Further deepening the analysis of physiological signal. Wang et al. developed high-speed surface electromyography (sEMG) monitors to accurately detect muscle activity onset and offset. They proposed two strategies—a threshold-based detector and a fast neural network-like detector—both utilizing a multi-resolution Teager-Kaiser energy operator for enhanced signal conditioning. Evaluations demonstrated that these energy operator-based monitors successfully suppressed noise interference, delivering superior change-point detection accuracy and high computational efficiency compared to standard statistical methods. Exploring pathological neuromuscular patterns, Zhang et al. proposed a high-density sEMG-based muscle force estimation framework to quantify hand dysfunction in children with cerebral palsy. They built a generalized LSTM network using healthy adult data, which was fine-tuned via transfer learning with healthy children’s data. The resulting gesture-specific networks achieved a root mean square error below 10%. Clinically, the abnormal EMG-force relationships observed in cerebral palsy patients correlated strongly with their clinical impairment grades. Finally, to facilitate adaptive control in human-in-the-loop exoskeleton systems, Liao et al. developed an identification method for human joint interactive torque using discrete sEMG signals. The authors applied principal component analysis to reduce the dimensionality of the sEMG features and utilized a backpropagation neural network to conduct regression learning within discrete joint angle intervals. This discrete learning approach was successfully converted into continuous torque profiles, achieving a mean squared error of 0.1502 across diverse motion states and providing a highly accurate feedback mechanism for intention-driven exoskeleton control.

## Biomechanical modeling and neuromuscular analysis

This section focuses on biomechanical modeling and neuromuscular analysis, providing deep insights into human motor control and its implications for advanced assistive technologies.

Wang et al. investigated neuromuscular control dynamics during sit-to-stand transitions. Utilizing non-negative matrix factorization and Gaussian graphical network modeling on EMG data, analysis revealed significant topological alterations in muscle synergy networks under robotic assistance. These findings suggest optimal assistance should preserve natural neuromuscular coordination networks rather than merely reducing biomechanical loads.

To translate models into real-time control, Li et al. addressed the computational challenges of joint torque estimation by proposing a sensitivity-based simplification method for a lower-limb musculoskeletal model. The team constructed an electromyography-driven knee joint model utilizing advanced Hill-type components for four major muscles and employed a genetic algorithm to identify individual-specific parameters. By applying Sobol’s global sensitivity analysis to evaluate the influence of parameter variations, they successfully isolated high-sensitivity variables to streamline the identification process. This methodical reduction effectively balanced the accuracy of torque estimation with computational efficiency, laying a robust foundation for practical human-machine interaction control in rehabilitation exoskeletons.

Expanding to interpersonal dynamics, Avaltroni et al. explored how haptic communication facilitates interpersonal coordination during hand-by-hand guided locomotion in both children and adults. The researchers captured comprehensive kinematic data and electromyographic activity from contact arm muscles to evaluate how physical touch dictates shared movement and dynamic muscle tone during gait initiation. Their results demonstrated that haptic interactions critically modulate muscle activity and locomotor control strategies across different dyads. These biomechanical insights into human-human physical coordination provide valuable paradigms for developing more intuitive, bio-inspired assistive technologies and refining pediatric gait rehabilitation interventions.

## Clinical rehabilitation strategies and functional training

Addressing critical clinical rehabilitation strategies, Ma et al. investigated targeted functional interventions for managing secondary lower extremity lymphedema in patients recovering from gynecologic cancer surgery. The researchers evaluated a 4-week rehabilitation protocol that integrated daily isokinetic strength training with standard manual lymphatic drainage. Their randomized controlled trial demonstrated that this combined intervention was significantly more effective than manual lymphatic drainage alone, leading to greater reductions in limb volume, enhanced calf muscle strength, and markedly improved walking ability.

Focusing on geriatric fall prevention, Zhu et al. investigated the immediate and retained effects of personalized reactive balance training on dynamic gait stability in older adults. They implemented a single-session perturbation-based program using unpredictable, progressively intense treadmill-induced slips and trips. The intervention significantly enhanced reactive balance control—specifically increasing the minimum margin of stability and reducing peak instability—both immediately and 3 months post-intervention, though it did not significantly alter the overall speed of recovery to steady walking.

## Summary

In summary, the research presented in this volume highlights a transition in rehabilitation engineering from passive motion therapies toward interactive, neuro-responsive, and personalized assistive technologies. The integration of electromyography (EMG) signal processing and multimodal intention recognition enhances human-robot interaction, enabling systems to dynamically adapt to users' neuromuscular states, joint torques, and physiological fatigue. Furthermore, adaptive, bio-inspired control strategies provide assist-as-needed support. This approach preserves natural biomechanical synergies and promotes active neural plasticity, rather than solely reducing biomechanical loads. Additionally, the focus on dynamic gait stability and reactive balance training reflects a clinical emphasis on functional resilience, aiming to reduce fall risks and improve real-world mobility. Ultimately, integrating biomechanics, advanced sensing, and responsive control offers promising pathways for advancing physical medicine. Continued cross-disciplinary innovation will be essential for developing seamlessly integrated robotics that effectively restore independence and improve patient quality of life.
